# Deep learning based solar forecasting for optimal PV BESS sizing in ultra fast charging stations

**DOI:** 10.1038/s41598-025-17408-0

**Published:** 2025-09-09

**Authors:** K. Lalbiakhlua, Subhasish Deb, Ksh. Robert Singh, Subir Datta, Hassan Abdurrahman Shuaibu, Taha Selim Ustun

**Affiliations:** 1 Department of Electrical Engineering, Mizoram University, Aizawl, Mizoram India; 2 Department of Electrical, Telecommunications and Computer Engineering, Kampala International University, Kampala, Uganda; 3 Fukushima Renewable Energy Institute, Koriyama, Japan

**Keywords:** Ultra-fast charging, Electric vehicles, Integrated charging station, Fast charging load demand, Energy infrastructure, Renewable energy, Electrical and electronic engineering

## Abstract

Ultra-fast charging stations (UFCS) present a significant challenge due to their high power demand and reliance on grid electricity. This paper proposes an optimization framework that integrates deep learning-based solar forecasting with a Genetic Algorithm (GA) for optimal sizing of photovoltaic (PV) and battery energy storage systems (BESS). A Gated Recurrent Unit (GRU) model is employed to forecast PV output, while the GA maximizes the Net Present Value (NPV) by selecting optimal PV and BESS sizes tailored to weekday and weekend demand profiles. The results show that PV integration alone improves the NPV by €6.19 million, while combining PV and BESS increases it to €33.97 million. With projected cost reductions, the system achieves a peak NPV of €34.05 million. Reliability analysis using Energy Sufficiency Ratio (ESR) and Autonomy Ratio (AR) confirms enhanced self-sufficiency and reduced grid dependency. This study demonstrates the techno-economic potential of hybrid renewable-powered UFCS using intelligent forecasting and evolutionary optimization.

## Introduction

The transportation sector is one of the largest contributors to global greenhouse gas (GHG) emissions, responsible for nearly 25% of global CO₂ output^1,2^. Despite improvements in industrial and power sector emissions, transport-related emissions have continued to rise, primarily due to fossil fuel dependency and increasing vehicle demand. Electric Vehicles (EVs) have emerged as a sustainable alternative, offering a promising solution to decarbonize transportation and reduce urban air pollution.

EV adoption has grown significantly, with the global EV market share rising from 2% in 2018 to 18% by 2023, and projected sales reaching 17 million units by the end of 2024^2^. To support this growth, scalable and high-performance charging infrastructure is essential. Ultra-Fast Charging Stations (UFCS), offering power outputs between 150 and 350 kW, can reduce charging times to under 30 min and are particularly crucial for long-distance travel and highway corridors^3^. However, UFCSs introduce operational challenges due to their high instantaneous power demand, which can lead to grid instability, increased capital costs, and heavy reliance on utility supply. Integrating UFCS with renewable energy systems—especially photovoltaic (PV) and battery energy storage systems (BESS)—has been proposed as a solution to reduce grid dependency, optimize energy usage, and improve sustainability^4^. Nevertheless, optimal sizing and coordination of PV and BESS remain complex, given the variability in solar generation, charging demand patterns, and battery degradation behavior^5,6^.

### Related work and research gap

Several studies have addressed the integration of renewable energy in EV charging stations^7–10^. For example^11^, proposed a multi-objective optimization model to maximize Net Present Value (NPV) in UFCS using PV and storage and the author in^12^, voltage stabilization strategy for UFCS operating within DC microgrids was implemented. The author in^13^ explored grid-integrated UFCS with energy storage, while^14^ examined hybrid wind-PV-BESS integration to enhance energy resilience in fast-charging stations. The author in^15^ developed an MILP formulation for storage-PV coordination in fast-charging stations. While these studies offer valuable insights, most rely on traditional forecasting techniques or static demand assumptions.

Genetic Algorithm (GA) is selected for optimization due to its global search capability, scalability to nonlinear objectives, and suitability for constrained techno-economic problems. Unlike Mixed-Integer Linear Programming (MILP), GA does not require convexity and handles discrete and continuous decision variables effectively. Compared to Particle Swarm Optimization (PSO) or Differential Evolution (DE), GA also offers better convergence control via its mutation and crossover strategies, making it suitable for multi-dimensional sizing problems in UFCS.

Despite advancements in hybrid PV-BESS system modeling, limited studies have combined deep learning-based solar forecasting with economic optimization for UFCS design. Deep learning-based approaches have been very popular recently^16^. Moreover, most existing models do not explicitly consider weekday and weekend demand variability or provide detailed profitability analysis using Net Present Value (NPV) as an objective function.

### Novelty

The novelty of this study lies in its integrated approach combining GRU-based deep learning for solar forecasting with NPV-driven optimization to size PV and BESS systems for an Ultra-Fast Charging Station (UFCS). Unlike previous studies that rely on static generation models or average demand assumptions, this work incorporates distinct weekday and weekend EV charging profiles using probabilistic vehicle arrival data. Additionally, the study introduces energy reliability metrics—Energy Sufficiency Ratio (ESR) and Autonomy Ratio (AR)—to assess grid independence and system resilience. This framework offers a realistic, data-driven, and economically robust solution for planning renewable-powered UFCS infrastructure under varying demand and evolving market conditions.

### Key contribution

This work proposes an integrated framework that combines deep learning-based solar forecasting with metaheuristic optimization for the design of renewable-powered Ultra-Fast Charging Stations (UFCS). The key contributions include:

- Implementation of Gated Recurrent Unit (GRU) networks for accurate PV generation forecasting.
- Modelling of realistic weekday and weekend EV charging demand using probabilistic vehicle arrival profiles.
- Optimization of PV and battery energy storage system (BESS) sizing using a Net Present Value (NPV) – driven Genetic Algorithm.
- Reliability evaluation through Energy Sufficiency Ratio (ESR) and Autonomy Ratio (AR); and.
- Sensitivity analysis considering future Capital Expenditure (CAPEX) reduction for PV and BESS.

These contributions collectively address the technical, economic and operational challenges of deploying intelligent, grid-resilient EV charging infrastructure.

## System design and methodology

This study considers an integrated Ultra-Fast Charging Station (UFCS) powered by a combination of photovoltaic (PV) panels, battery energy storage system (BESS), and the utility grid. The system is designed to reduce reliance on grid energy, minimize operational costs, and ensure a reliable power supply for electric vehicles (EVs), even during periods of solar intermittency or peak demand. A DC-coupled configuration is adopted for efficient energy conversion and simplified integration. The components are interconnected through a DC bus to minimize AC/DC conversion losses and to enable seamless operation between renewable sources, energy storage, and charging points^11^.

- Grid: Connected via a bidirectional AC/DC inverter, allowing energy flow to/from the grid.
- Solar PV: Connected through a DC/DC converter to supply renewable energy directly to the DC bus.
- BESS: Linked via a bidirectional DC/DC converter for energy storage and discharge to balance demand.
- DC Bus: Serves as the central hub, minimizing conversion losses and providing stable DC power.
- EV Charger: Draws power directly from the DC bus for efficient and fast charging.

In this application, vehicle-to-grid technology will not be considered. In order to avoid requiring EV users to supply power to the grid, ultra-fast charging stations are designed to charge batteries as quickly as feasible. Thus, installing an ultra-fast charging station is the best option for quick charging because of its higher power, which may range from 150 kW to 300 kW and guarantees a full charging period of 15 to 35 min.

**Fig. 1:**
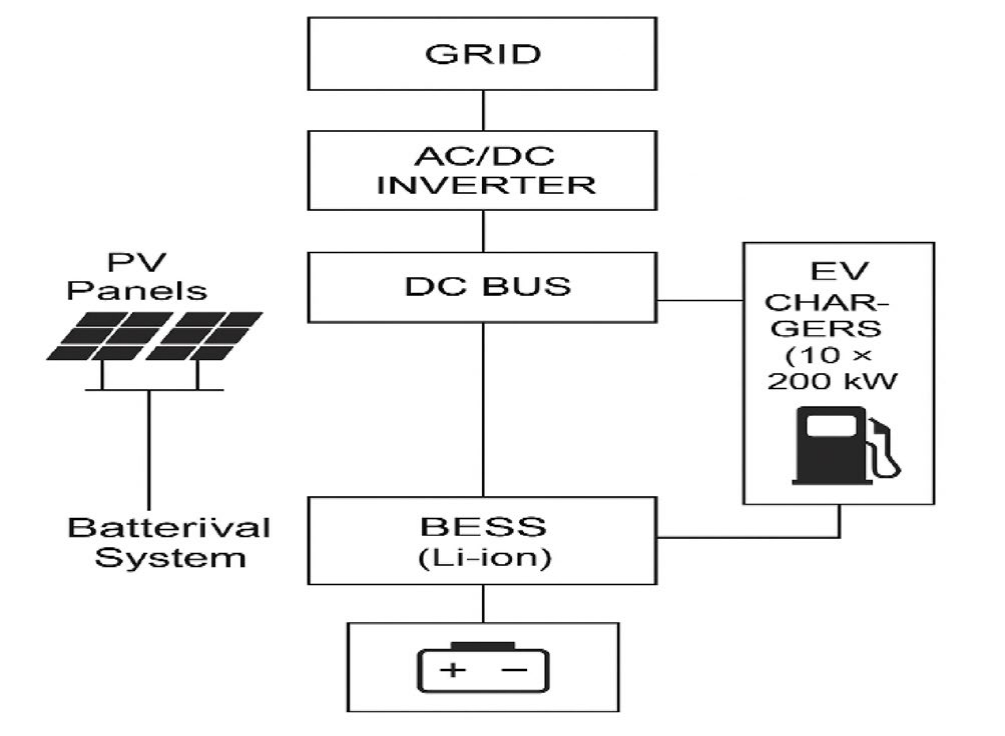
System architecture of the proposed ultra-fast EV charging station integrating photovoltaic (PV), battery energy storage system (BESS) and grid via DC bus.

The overall system structure is illustrated in Fig. 1. The proposed design ensures energy prioritization from PV, stores excess energy in the BESS, and uses grid power only when necessary. The energy management strategy is further elaborated in subsequent sections, including the charging demand profile, forecasting, and optimization.

### Charging demand

#### Electric vehicle arrival time

**Fig. 2:**
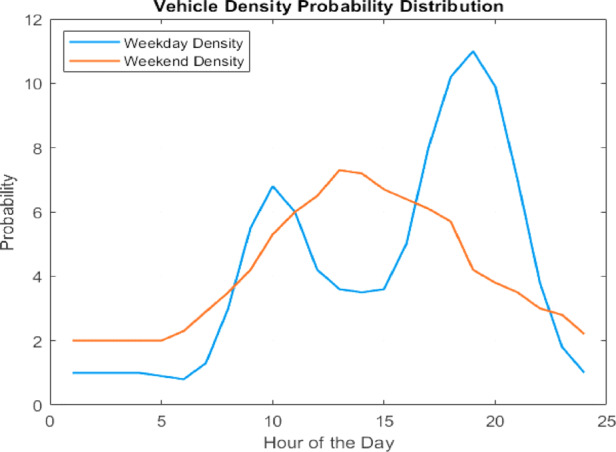
Vehicle Density probability distribution.

To estimate the daily energy requirement of EVs at the UFCS, probabilistic vehicle arrival data from the Italian National Road Authority^18^ was used. This data, specific to a highway service area, provides the likelihood of vehicle arrivals throughout the day and is visualized in Fig. 2 as a probability distribution curve. This distribution reflects realistic traffic flow patterns and is crucial for accurately modelling charging demand.

#### Electric vehicle categories

The EV fleet is divided into six categories: small, medium, and large passenger cars; trucks; buses; and electric motorcycles. Their proportions vary between weekdays and weekends, based on EV market data from Italy^20^. Table 1 shows the percentage share of each vehicle category for both day types.

**Table 1:** Vehicle fleet share^18^.

Days	Cars	Heavy vehicles	Bikes (10% EV)
Weekdays	15% Large, 5% Medium, 3% Small	75% 10% buses, 90% trucks	2%
Weekends	15% Large, 10% Medium, 7% Small	65% 100% buses	3%

#### Electric vehicle charging profiles

“Charging profiles were obtained from Fastned, based on real-world curves of popular EV models in Italy.”^19^. Charging profiles are selected from actual fast-charging curves of best-selling EVs in Italy^20^. Each profile includes battery capacity and maximum charging rate:

- Small: Fiat 500e, battery 42 kWh,
- Medium: Tesla model Y, battery 60 kWh,
- Large: BMW iX4, battery 77 kWh.

**Fig. 3:**
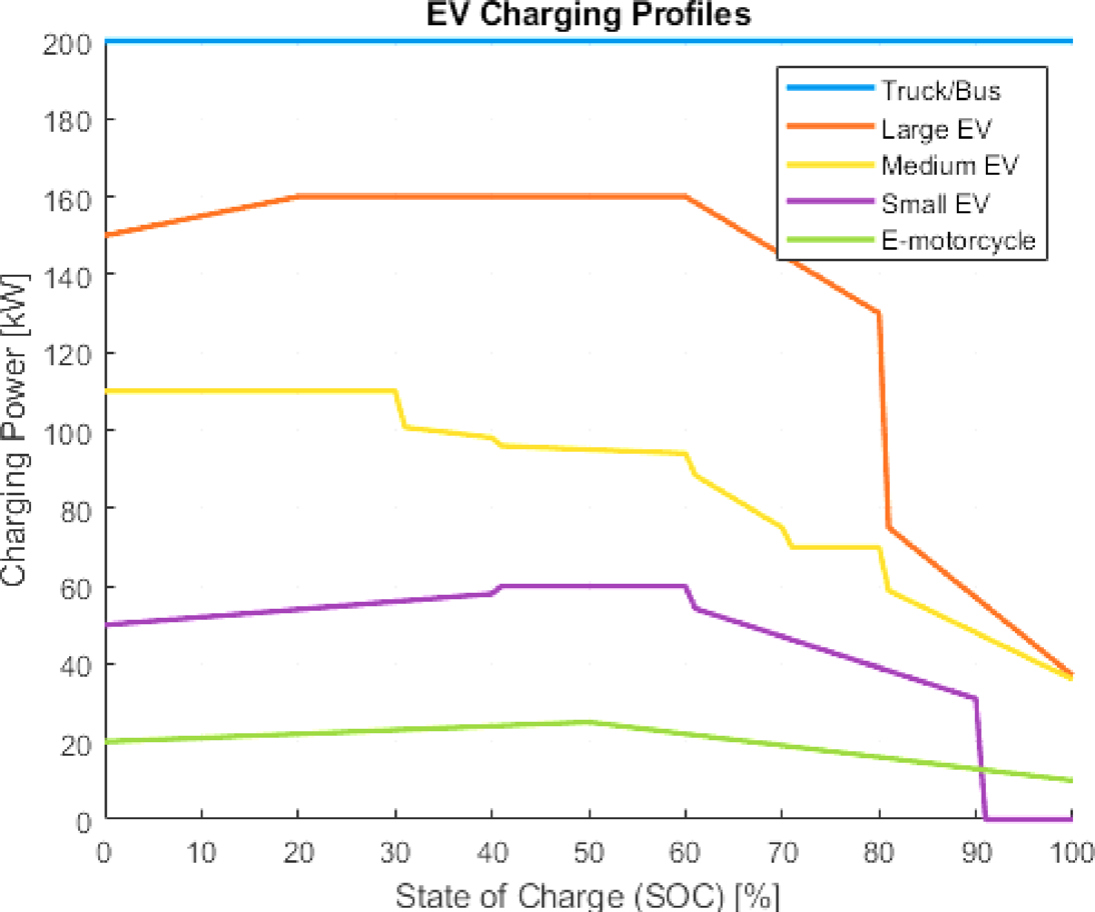
Charging profiles for different EV^19^.

The bike has a battery is taken as 22 kWh as assumption and for truck/bus with a battery capacity taken as 250 kWh with a constant charging power of 200kW^3^,, is considered in Fig. 3.

#### State of charge (SOC) at arrival

The SoC of an Electric Vehicle (EV) arriving at the Ultra-Fast Charging Station (UFCS) is modelled using a Weibull distribution, given by^21^:

1 $${f_{SOC}}(SOC;a,b)=\frac{b}{a}{(\frac{{SOC}}{a})^{b - 1}}{e^{ - {{(\frac{{SOC}}{a})}^{^{b}}}}}$$

**Fig. 4:**
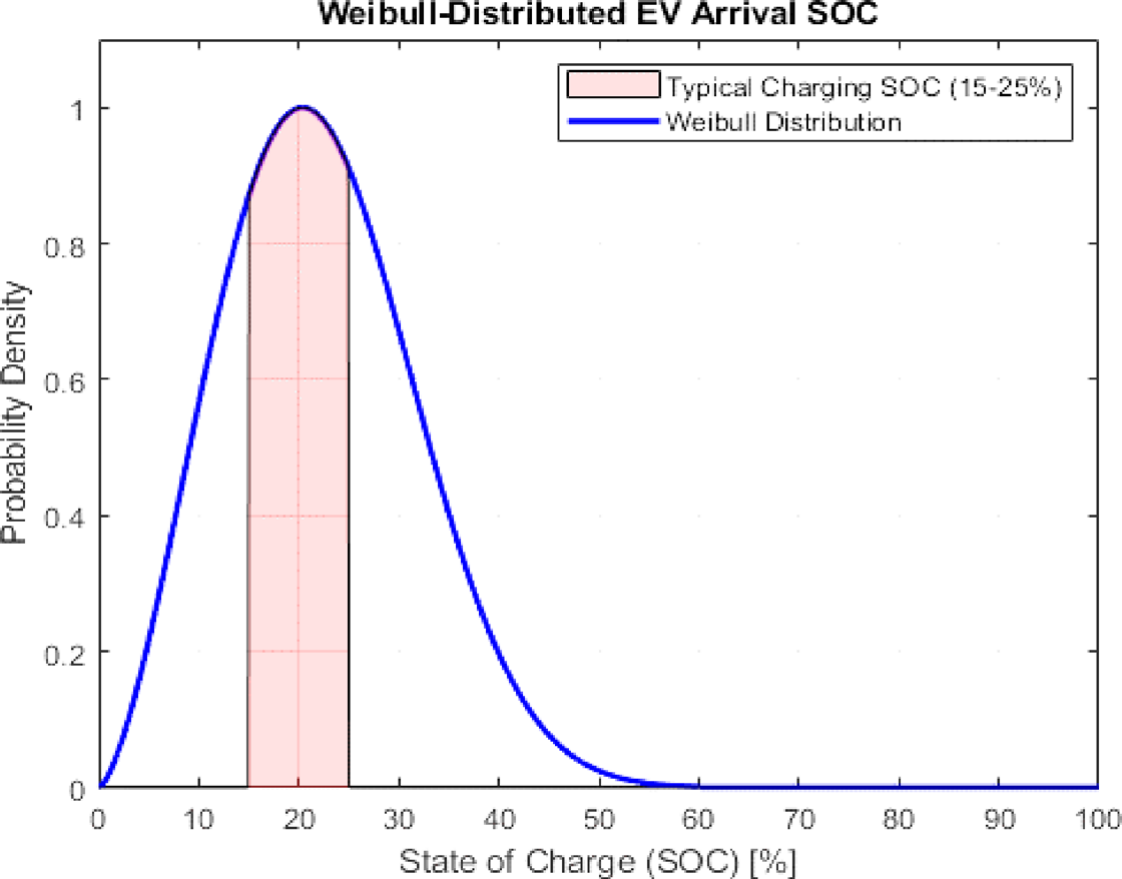
Electric Vehicle Arrival State of Charge.

The distribution peaks around 20% SoC with parameters a = 2.4 and b = 2.5, indicating that drivers are most likely to charge while their battery is between 15% and 25%. Over 60% SoC, the likelihood of stopping at the station drops significantly. This model aids in operational optimization and charging station traffic prediction. The given Weibull distribution is illustrates in Fig. 4.

#### Charging station

**Fig. 5:**
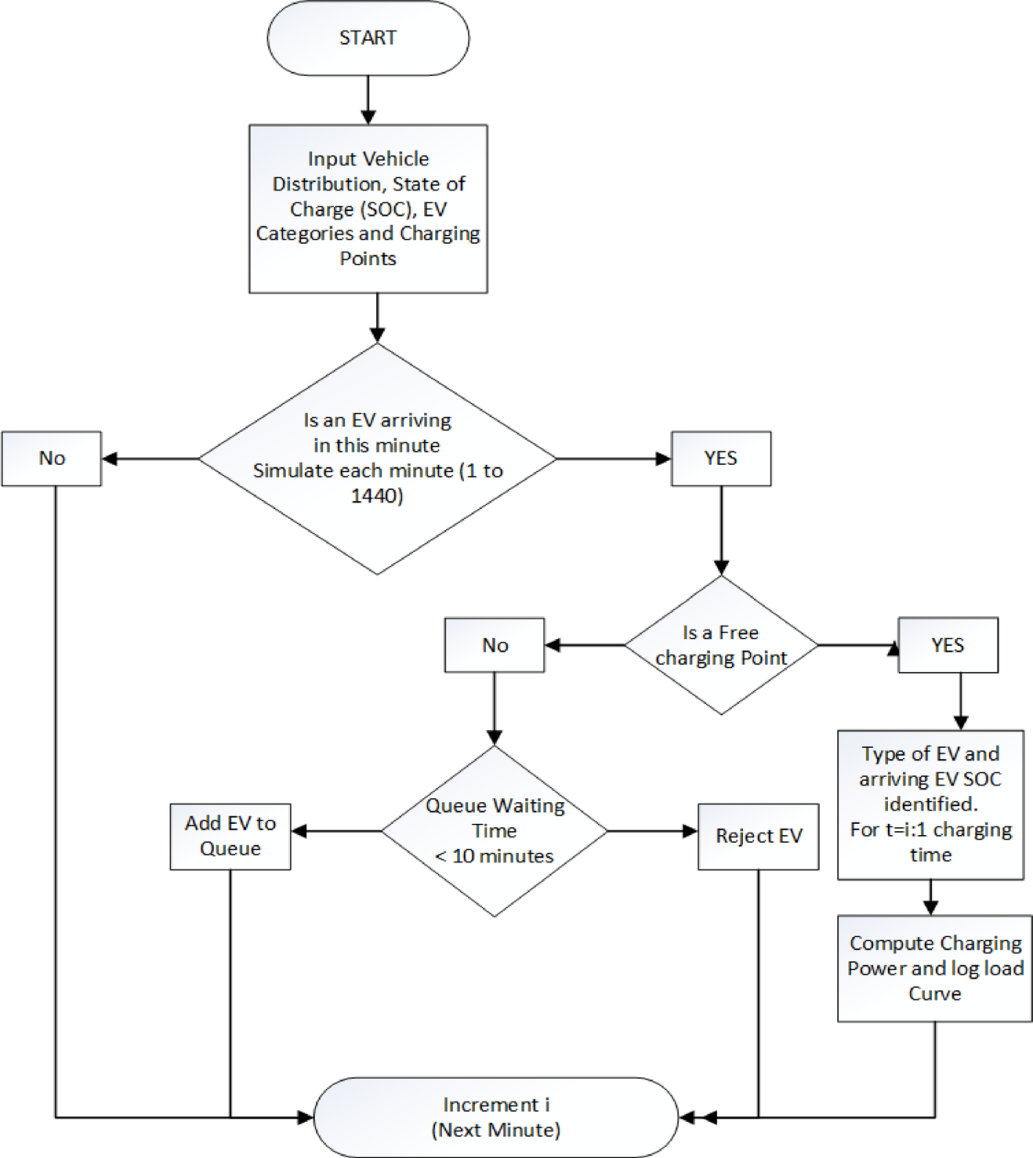
Flowchart for EV Charging Demand Simulation Logic over 24 h.

**Fig. 6:**
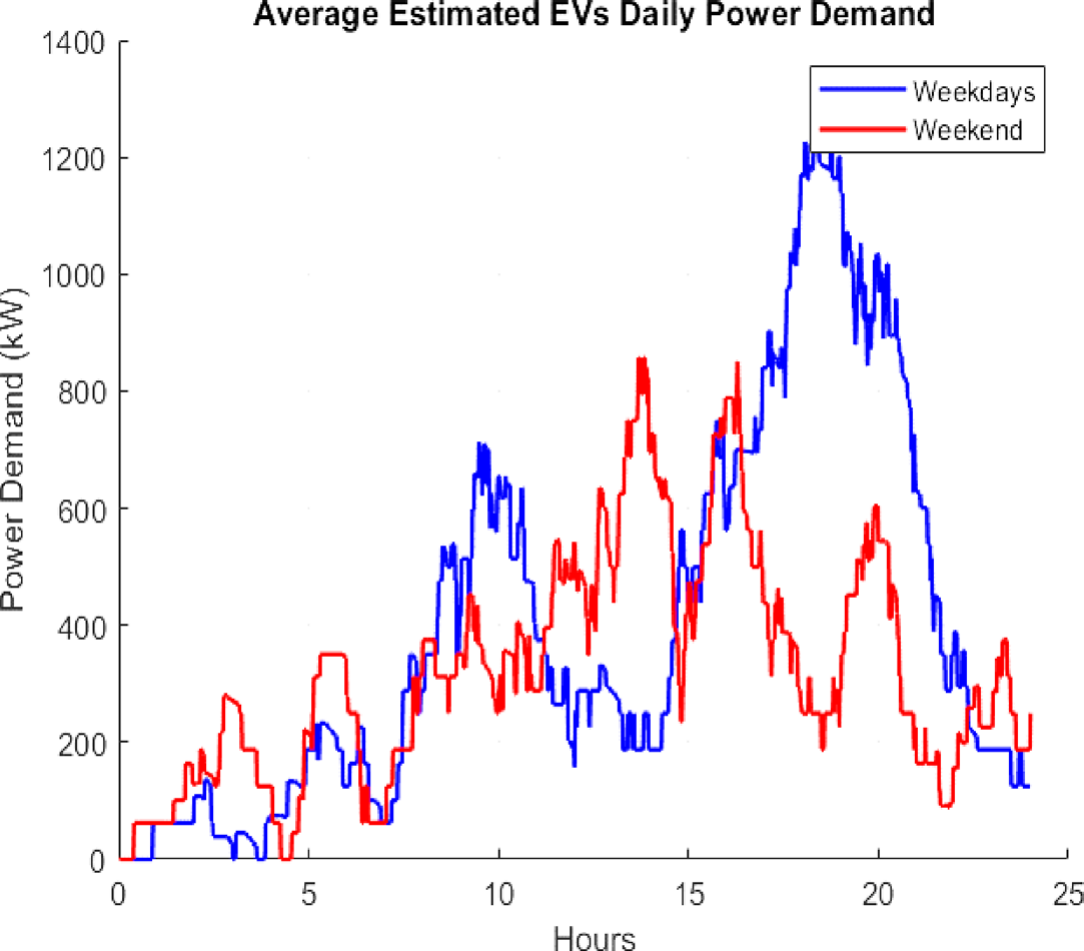
Power Demand Curve.

With a maximum waiting line time of 15 min, an ultra-fast charging station of 10 charging points with 200 kW each is taken into consideration. The charging demand for the charging station is determined by following the procedures in Fig. 5, and it is displayed in Fig. 6 on a weekend and weekday basis. Weekdays have a higher power demand because there are more automobiles available during these times. Approximately 3332.49 MWh of electricity are used annually by the charging station. The flowchart Fig. 5 outlines the operational logic for managing electric vehicle (EV) charging at a station over a 24-hour period, broken into 1,440 min. The detailed operational steps are described below.

1. Initially, the model inputs key parameters, including the vehicle distribution across various categories (e.g., cars, trucks, buses, bikes), the state of charge (SOC) distribution at arrival, and the number of available charging points.
2. The simulation is executed for each of the 1,440 min in a day. At each time step, the system evaluates whether an electric vehicle (EV) arrives based on probabilistic arrival data. If no EV arrives, the simulation advances to the next minute.
3. When an EV arrives, the system checks for the availability of a free charging point. If no charger is available, the algorithm assesses the waiting time in the queue. If the queue waiting time is less than 10 min, the EV is added to the queue; otherwise, it is rejected from the system.
4. If a charging point is available, the arriving EV is accepted, and its type (e.g., small car, medium car, truck, etc.) and initial SOC are identified. The charging session is then simulated over its duration, with charging power computed and the corresponding demand logged at each time interval.
5. This process is repeated iteratively for every minute of the day, ensuring accurate modeling of EV arrival, queuing, charging, and power demand. The final output is a detailed load profile that represents the total energy consumption of the UFCS over a 24-hour period, segregated by weekdays and weekends.

### System components

#### Photovoltaic system model

For the PV power production, historical solar irradiance and environmental data, including Global Horizontal Irradiance (GHI), Direct Normal Irradiance (DNI), and temperature, were taken from NSRDB: National Solar Radiation Database^24^. They are used to train the models. The PV output is calculated using formula given in (2):

2 $$\begin{aligned} & PV{\text{ }}Output{\text{ }}\left( W \right){\text{ }} = {\text{ }}Plane{\text{ }}of{\text{ }}Array{\text{ }}Irradiance{\text{ }}\left( {W/m^2} \right){\text{ }} \times \hfill \\ & {\text{ }}Panel{\text{ }}Efficiency{\text{ }}x{\text{ }}Panel{\text{ }}Area{\text{ }}\left( {m^2} \right) \hfill \\ \end{aligned}$$

### Forecasting techniques

**Fig. 7:**
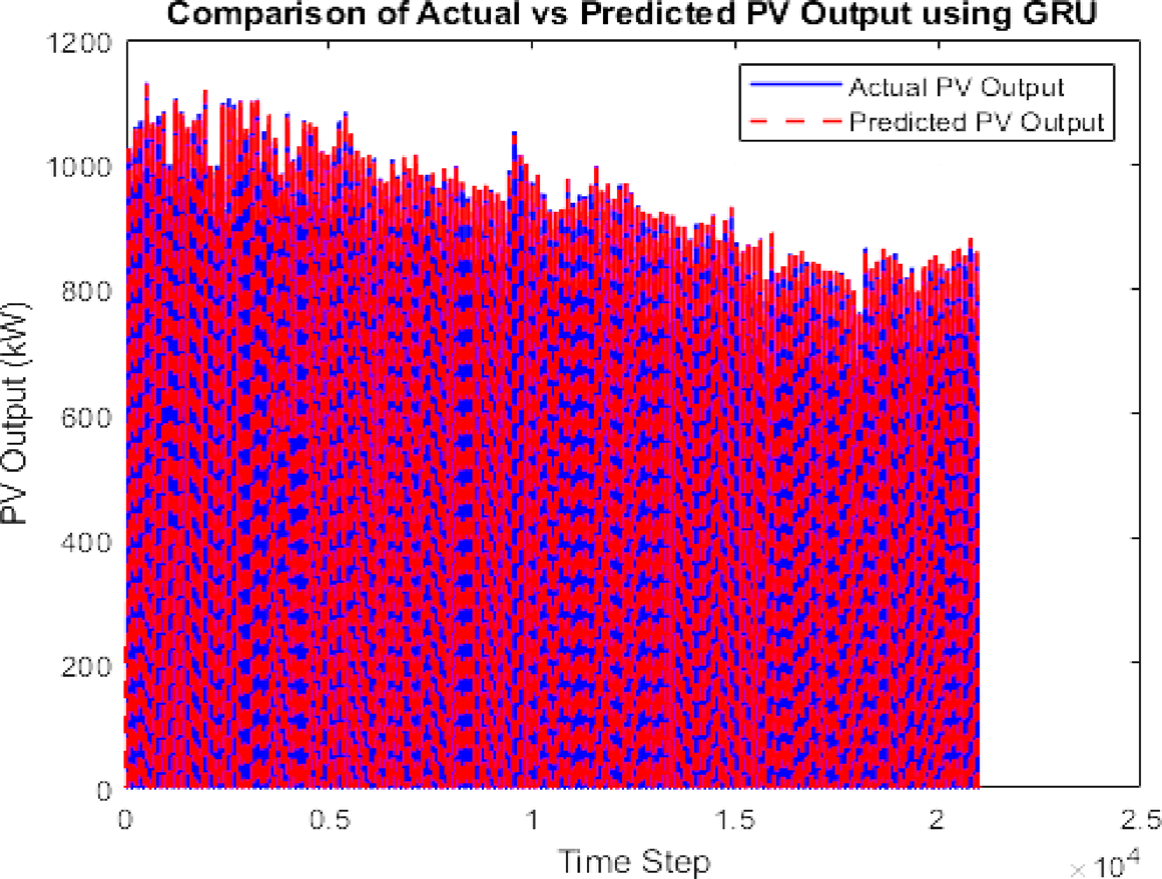
GRU forecasted PV.

**Fig. 8:**
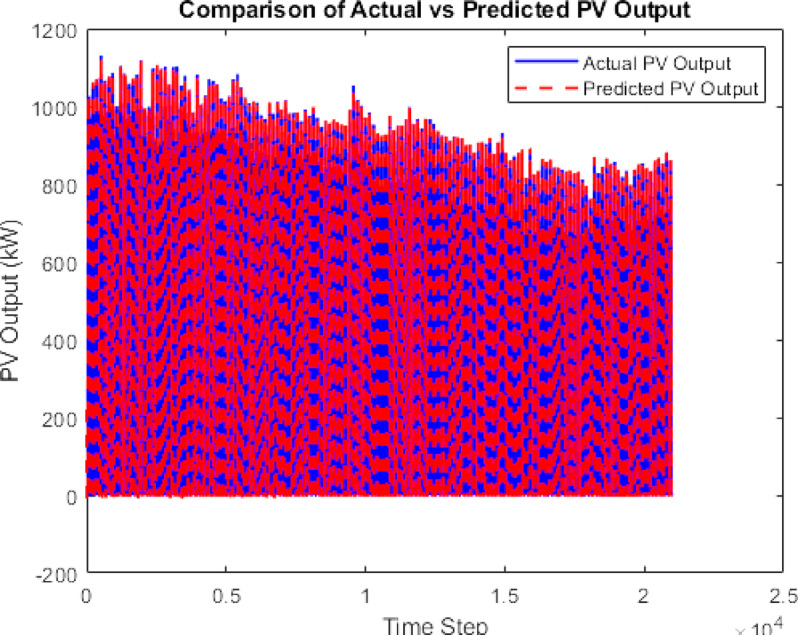
LSTM Forecasted PV.

The data is then forecasted using a deep learning tool. It is a kind of machine learning model that draws inspiration from the human brain’s architecture. “The Gated Recurrent Unit (GRU) model, known for its computational efficiency and capability to capture temporal dependencies, was selected for PV forecasting.”^23^. Using the LSTM and GRU the PV output is being forecasted given in Figs. 7 and 8. A comparison with LSTM showed better accuracy for GRU using error analysis in this dataset.

### Battery energy storage system

For the charging station, a lithium-ion battery energy storage system is taken into consideration. The discharge efficiency (ηdisBESS) and charging efficiency (ηchBESS) are assumed to be 92% and 96%, respectively. The battery pack’s restricted lifespan of 3000 is determined as in (3).

3 $${N_{cycle}}=\sum\limits_{{d=1}}^{{365}} {\sum\limits_{{t=1}}^{{1440}} {\frac{{|SO{C_t} - SO{C_{t - 1}}|}}{{2 \times DoD}}} }$$

Where, DoD is depth of discharge, SOC is the state of charge respectively. The battery operates within 10–90% SOC, with a depth of discharge (DoD) of 80%. It is assumed to have a 2-hour discharge duration (C-rate = 0.5 C).

## Optimization formulation

### Objective problem

The goal of this effort is to maximize system profitability, and the UFCS’s size will be influenced by economic factors. Therefore, if the price of energy sold to EV customers is 70 c€/kWh and the price of energy received from the grid is 17.46 c€/kWh, the grid’s selling price can be regarded as one-tenth of the price paid for the energy.

The NPV of the system can be calculated given as in (4):

4 $$\begin{gathered} NPV= - CAPE{X_k}.{P_k} - \operatorname{Re} p{l_k}.{E_k}+ \hfill \\ \,\,\,\,\,\,\,\,\,\,\,\,\,\,\,\,\sum\limits_{{year=1}}^{{lifetime}} { \times \frac{{CashI{n_{yearly}} - CashOu{t_{yearly}} - OPE{X_k}.{P_k}}}{{{{(1+r)}^{year}}}}} \hfill \\ \end{gathered}$$

Where,

- P_k_ is power of the k^th^ component.
- E_k_ is energy capacity.
- Repl_k_ is replacement cost.
- CashIn_yearly_ is the yearly income earned.
- CashOut_yearly_ is the yearly expenses.
- r is discount rate.
- OPEX is operating expenditure.

The numerical used can be given with considering discharging rate of 0.5 (2 h) in the given Table 2.

**Table 2:** Numerical values.

Parameter	Value
CAPEX_PV_	1000 €/kW
OPEX_pv_	160 €/kW/year
R	2.5%
CAPEX_BESS_	439 €/kWh
Repl_BESS_	196 €/kWh
Lifetime	20
OPEX_BESS_	4.28 €/kWh/year

#### Constraints

The optimization problem is subject to the following constraints:

i) **Energy Balance**: At every time step, the energy balance must be satisfied:

5 $$\begin{gathered} Demand(kW)=PV\,Output(kW)+BESS\,DISCHARGE(kW) \hfill \\ +Grid\,Energy(kW) \hfill \\ \end{gathered}$$

Where, Energy Demand at UFCS ≤ Energy Supply from PV + BESS.

ii) **Battery charging/discharging Exclusivity**: The battery cannot charge and discharge simultaneously.

6 $$BESS\,Ch\arg e(kW).BESS\,Disch\arg e(kW)=0$$

iii) **The battery SOC remains within a specified limit**:

7 $$SO{C_{min}} \leq SOC{\text{ }}\left( t \right) \leq SO{C_{max~~}}$$

iv) **Charging/Discharging power must respect BESS limits**:

8 $$0 \leq BESS{\text{ }}Charge{\text{ }}\left( {kW} \right) \leq {P_{BESS,{\text{ }}max~~~~~}}$$

9 $$0 \leq BESS{\text{ }}Discharge{\text{ }}\left( {kW} \right) \leq {P_{BESS,{\text{ }}max}}$$

v) **Operational Constraints**:

- Ensure that the BESS operates within its charge and discharge limits,
- Ensure that the PV panels operate within expected solar irradiance levels.

#### Reliability test

The reliability of the UFCS system is evaluated using two key metrics: Energy Sufficiency Ratio (ESR) and Autonomy Ratio (AR). These metrics assess the system’s ability to operate efficiently and independently from the grid.

- **Energy Sufficiency Ratio (ESR)**: The Energy Sufficiency Ratio measures how much of the total energy demand is met without relying on grid energy. It is expressed as a percentage and is calculated using the formula:

10 $$ESR=(1 - \frac{{TotalGridEnergyUsed}}{{TotalEnergyDemand}}) \times 100$$

Where, total Grid Energy Used is the sum of all energy drawn from the grid to meet deficits over the evaluation period.

- **Autonomy Ratio (AR)**: The Autonomy Ratio evaluates the percentage of time intervals (time steps) where the UFCS operates without using energy from the grid. It is computed using the formula and given as a percentage:

11 $$AR=(\frac{{NumberofTimeStepswithoutGridUsage}}{{TotalNumberofTimeSteps}}) \times 100$$

Where, Number of Time Steps without Grid Usage is the count of time intervals where the net energy supply from PV and BESS is sufficient to meet the UFCS demand without requiring grid energy.

## Optimization workflow

**Fig. 9:**
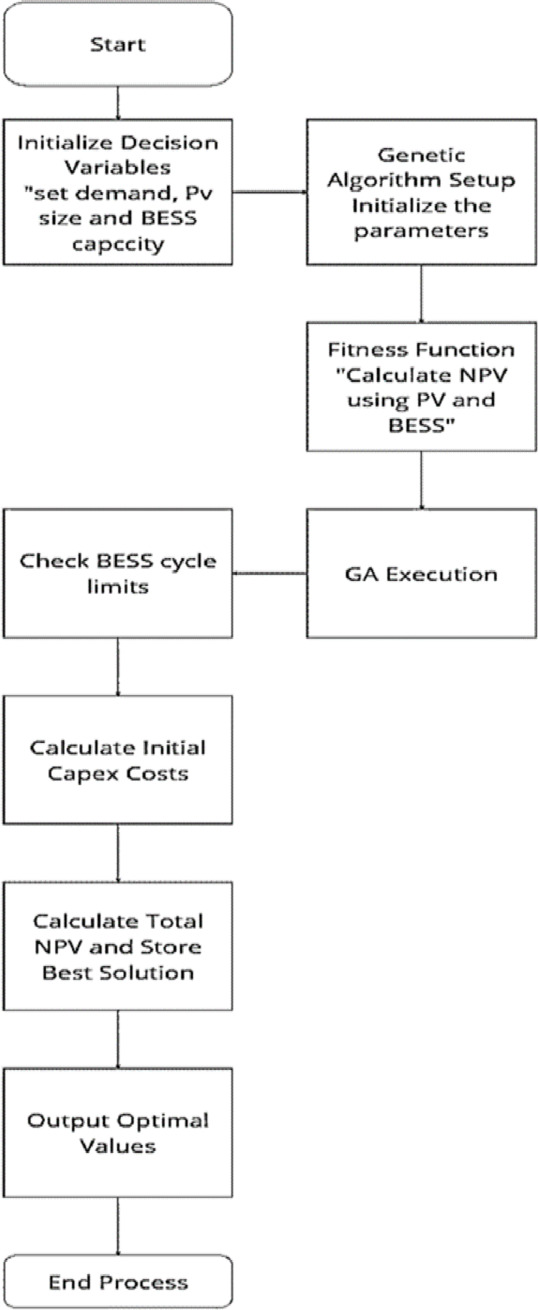
Optimization flowchart for maximizing the NPV using GA.

The overall optimization process is illustrated in Fig. 9. The algorithm begins by defining the input parameters, including:

- Forecasted energy demand (weekday/weekend).
- PV generation (from deep learning forecasts).
- Technical and economic specifications of PV modules, BESS, and grid interaction.
- Financial metrics such as capital and operational costs, discount rate, and energy prices.

The decision variables are the PV system size (kWp) and BESS energy capacity (kWh). A Genetic Algorithm (GA) is employed to search for the optimal configuration. GA parameters include population size, number of generations, and crossover/mutation probabilities.

Each candidate solution is evaluated using a fitness function that simulates hourly energy flows over a one-year horizon. The function computes:

- PV generation and load satisfaction.
- BESS charging/discharging with SOC tracking and efficiency losses.
- Grid imports and exports.
- Revenues from EV charging and associated costs.

The NPV is calculated using discounted cash flow analysis for each solution. The GA iteratively evolves the population until convergence or the maximum number of generations is reached.

### PV-Only integration scenario

In the first configuration, only a PV system is integrated into the UFCS. The Genetic Algorithm searches for the optimal PV size that maximizes profitability. The system operation logic is as follows:

- If PV generation > demand: the surplus is either curtailed or sold to the grid at a low feed-in tariff.
- If demand > PV generation: the remaining energy is purchased from the grid at a higher cost.

### Scenario 2: PV and BESS integration

Photovoltaic panel and Battery energy storage integration.

In the second configuration, both PV and BESS systems are integrated. The GA simultaneously optimizes both PV and battery capacities. The goal is to:

- Increase self-consumption of solar energy.
- Reduce energy procurement from the grid.
- Store excess solar energy during low demand periods and dispatch it during peak demand.

System operation rules in this scenario:

- If PV generation > demand:

Charge the BESS up to its capacity.

Sell or curtail any excess energy once the BESS is full.

- If demand > PV generation:

Discharge the BESS if SOC allows.

If the BESS is depleted, import energy from the grid.

- BESS Operation:

Charging/discharging efficiencies are considered (e.g., 96% charge efficiency, 92% discharge efficiency).

SOC is constrained between 10% and 90% of capacity, based on a Depth of Discharge (DoD) of 80%.

The BESS lifetime is modeled with a cycle limit and replacement costs factored into NPV.

This configuration improves energy autonomy and economic performance by shifting energy availability to match peak demand periods.

### BESS characteristics and comparison with conventional ESS

Battery Energy Storage Systems (BESS), especially lithium-ion types, are favored for UFCS due to their fast response, high energy density, and modular design^23^. Unlike conventional ESS (e.g., pumped hydro or flywheels), BESS offers better space efficiency and quicker deployment. However, they come with higher upfront costs and degrade over time with cycling. Despite this, their performance and flexibility make them ideal for high-demand, space-limited EV charging setups.

### Forecasting and GA justification

To improve prediction accuracy of PV generation, both Long Short-Term Memory (LSTM) and Gated Recurrent Unit (GRU) neural networks were tested. GRU achieved lower RMSE and faster convergence, making it the preferred forecasting model.

Genetic Algorithm was chosen due to its flexibility in handling:

- Mixed variable types (discrete PV size, continuous BESS capacity).
- Nonlinear constraints (e.g., SOC dynamics, charging curves).
- Multiple economic objectives (revenue vs. cost trade-offs).

## Result and discussion

The results present a comprehensive analysis of the simulation and optimization results for the Ultra-Fast Charging Station (UFCS). Three system configurations are evaluated—grid-only (baseline), PV-only, and PV integrated with a Battery Energy Storage System (BESS). The optimization of each configuration is conducted using a Genetic Algorithm (GA) to maximize the Net Present Value (NPV). Demand forecasting is carried out using a Gated Recurrent Unit (GRU)-based deep learning model, and performance is assessed through MATLAB-based simulations.

### Baseline scenario: Grid-Only UFCS

The initial scenario involves operating the UFCS entirely using grid-supplied electricity. The forecasted average daily demand is 9669.52 kWh on weekdays and 7776.41 kWh on weekends, totaling 3,332,490.84 kWh annually. The first-year revenue generated from EV charging is estimated at €2,332,743.58, while the annual cost for purchasing electricity from the grid is €581,852.90. Over a 20-year analysis period, the baseline NPV is determined to be €27.29 million, serving as a reference for further evaluations.

### Scenario 1: PV integration

In this configuration, PV energy is used to supplement grid power. The Conergy PowerPlus 300 Wp monocrystalline modules are employed for simulation. Using GRU-based forecasts, August 13 (weekday) and August 9 (weekend) are selected as representative days for evaluating PV performance. The results show:

- Surplus PV energy: 90,462.30 kWh (weekday) and 78,070.07 kWh (weekend).
- Grid energy purchased: 166,045.23 kWh (weekday) and 96,859.92 kWh (weekend).

**Fig. 10:**
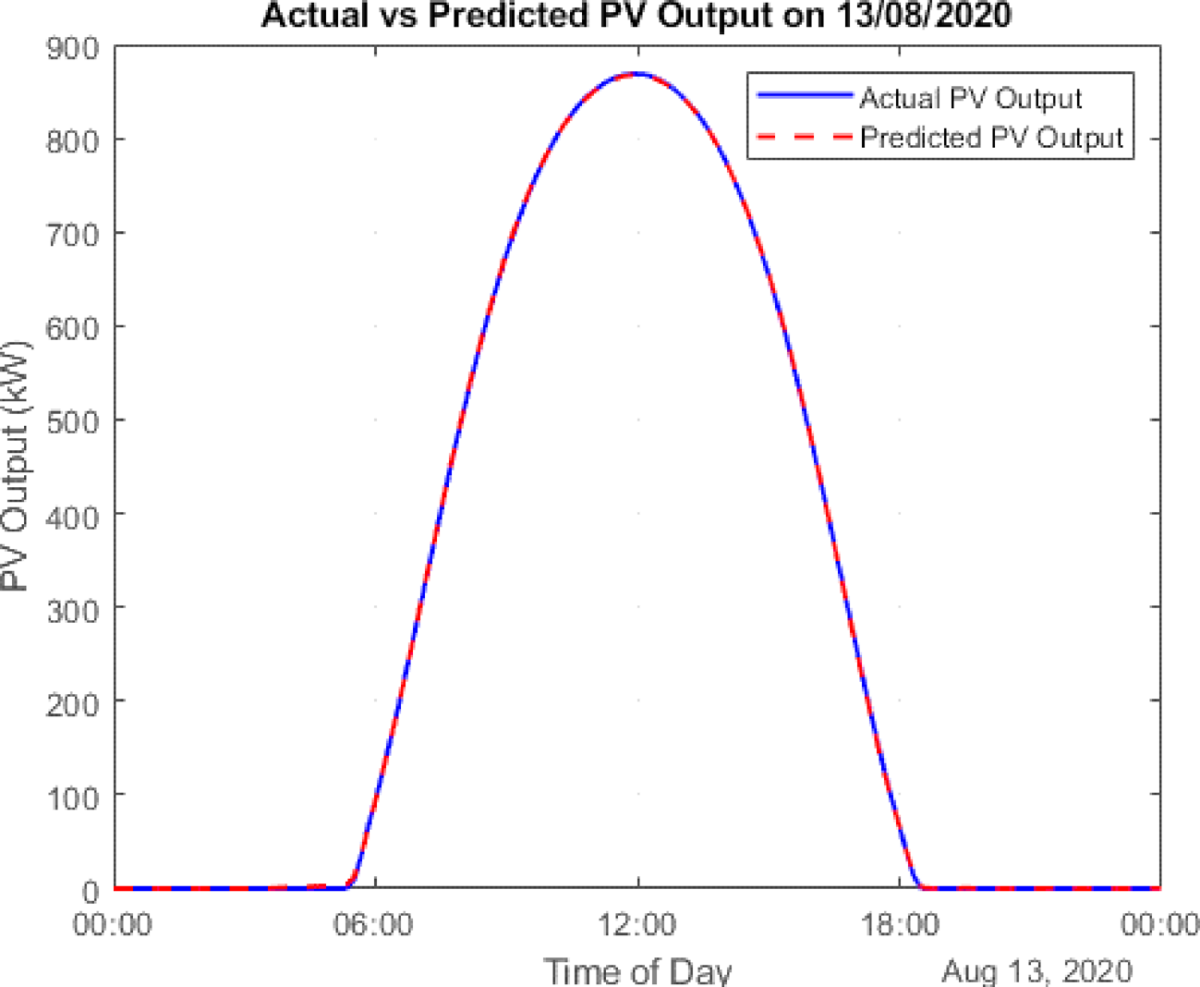
Forecasted PV power output for August Weekday (13th Aug 2020).

**Fig. 11:**
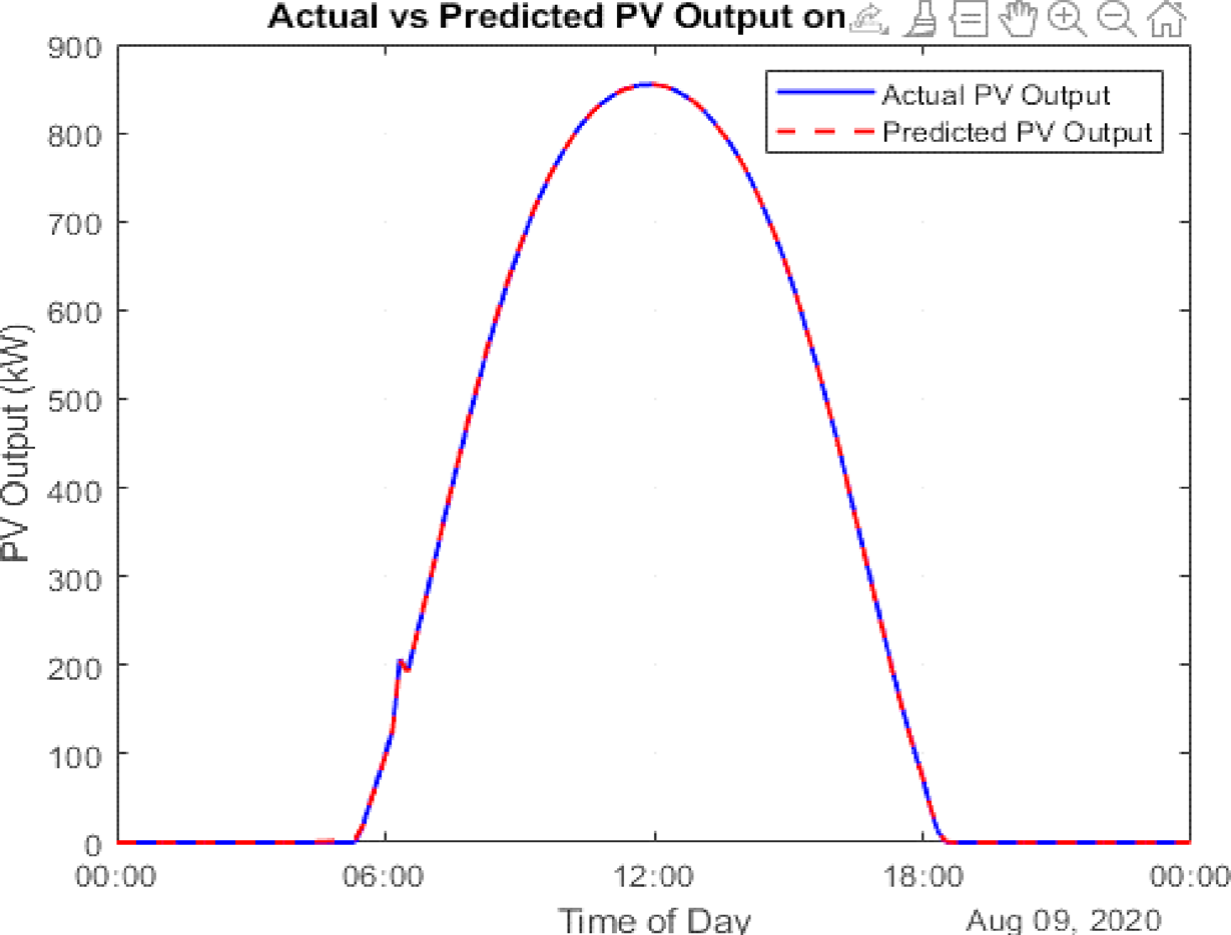
Forecasted PV power output for August Weekend (9th Aug 2020).

**Fig. 12:**
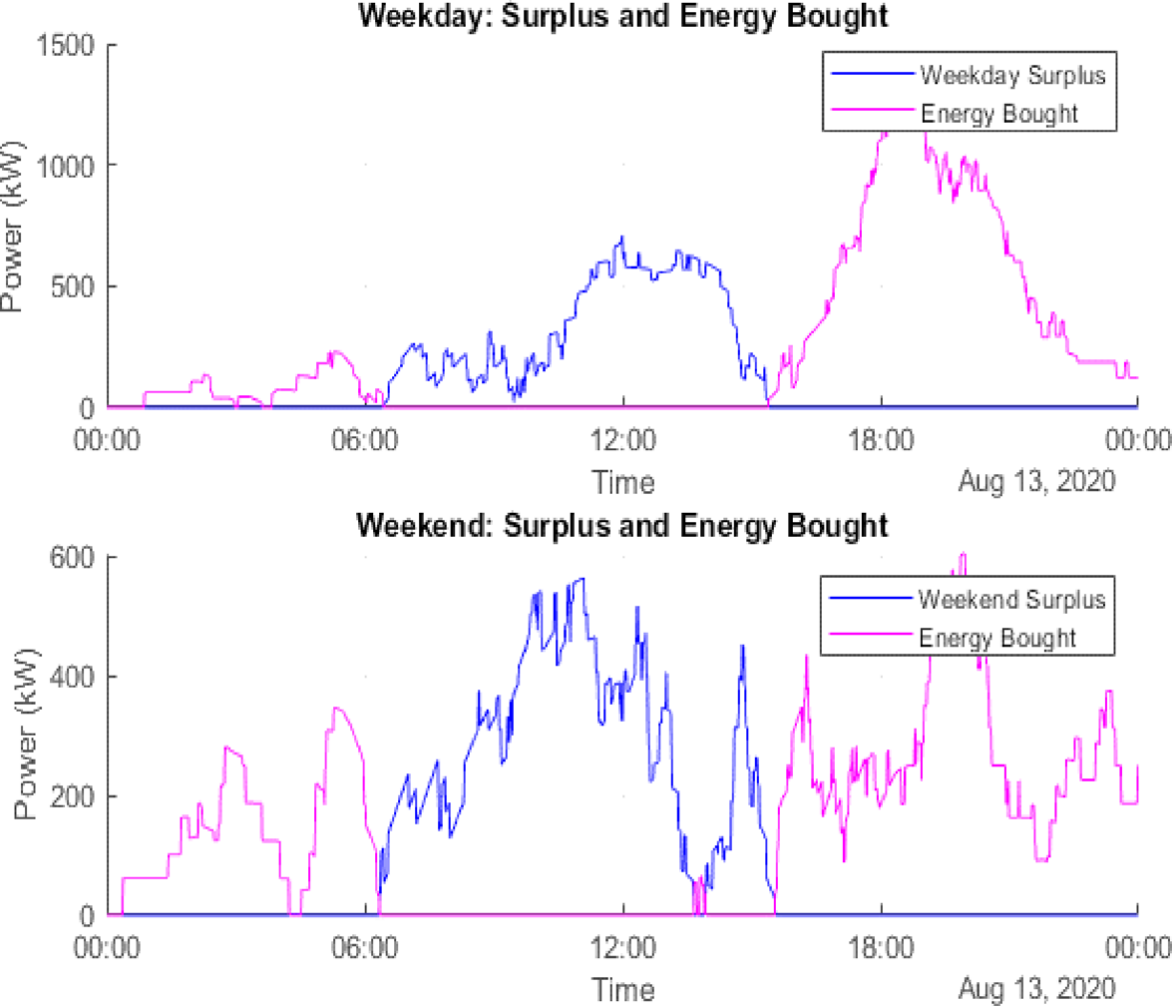
Power Surplus of PV and Demand.

Figure 10 illustrates the PV generation and load demand on August 13 (weekday), while Fig. 11 provides a similar comparison for August 9 (weekend). The actual PV output value of solar data has been taken from the National Solar Radiation Database at a specific location in Italy^24^. Figure 12 presents the cumulative surplus and grid energy purchases for both scenarios. These results highlight improved energy self-sufficiency, particularly on weekends, though considerable surplus PV energy remains unutilized.

### Scenario 2: PV and BESS integration

This hybrid configuration addresses PV surplus losses by incorporating a BESS to store excess energy for later use. Simulation outcomes reveal:

- Battery-stored energy: 358,308.80 kWh (weekday) and 354,529.78 kWh (weekend).
- Remaining surplus energy: 89,956.64 kWh (weekday) and 77,213.23 kWh (weekend).
- Grid energy deficit: 165,598.63 kWh (weekday) and 96,103.16 kWh (weekend).

**Fig. 13:**
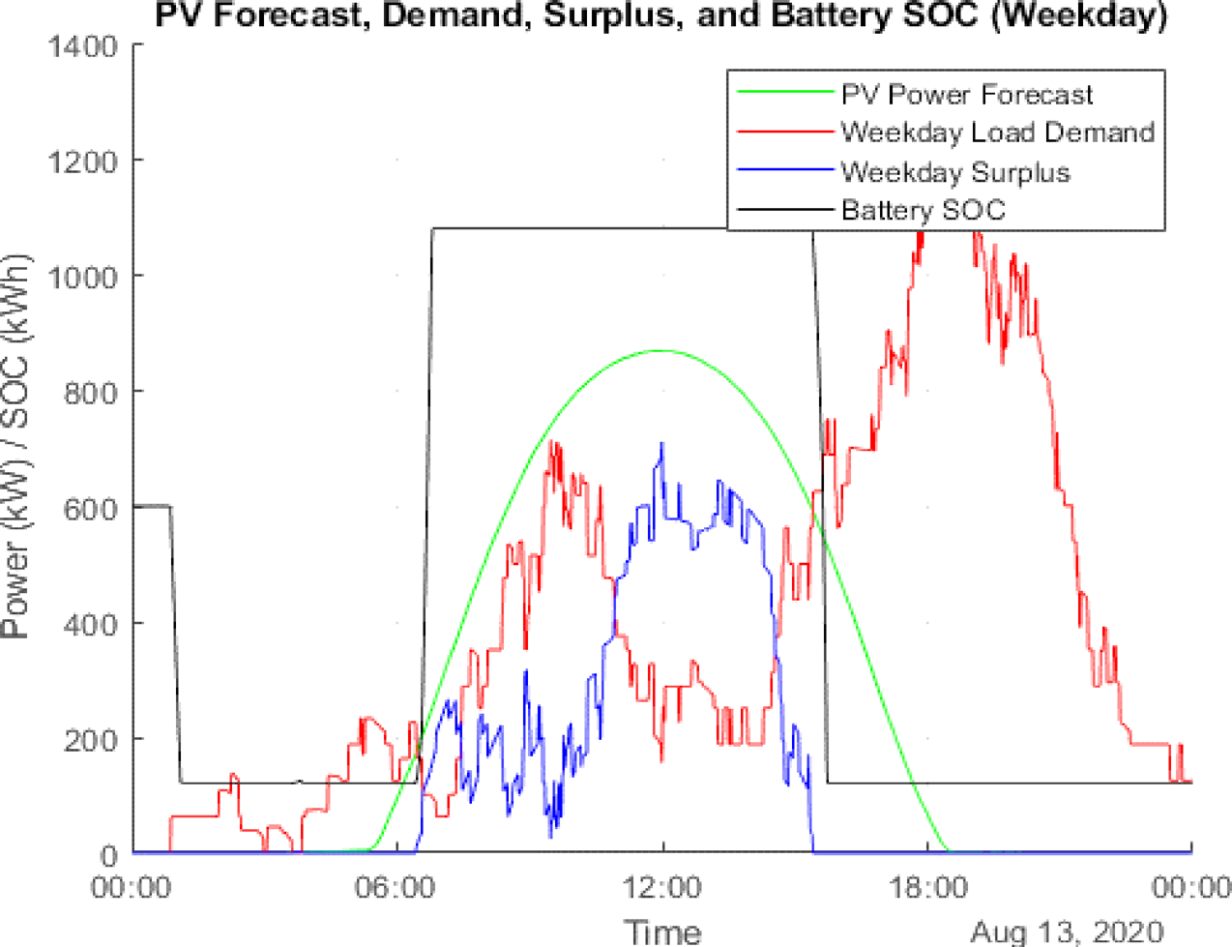
Weekday power surplus with PV and BESS.

**Fig. 14:**
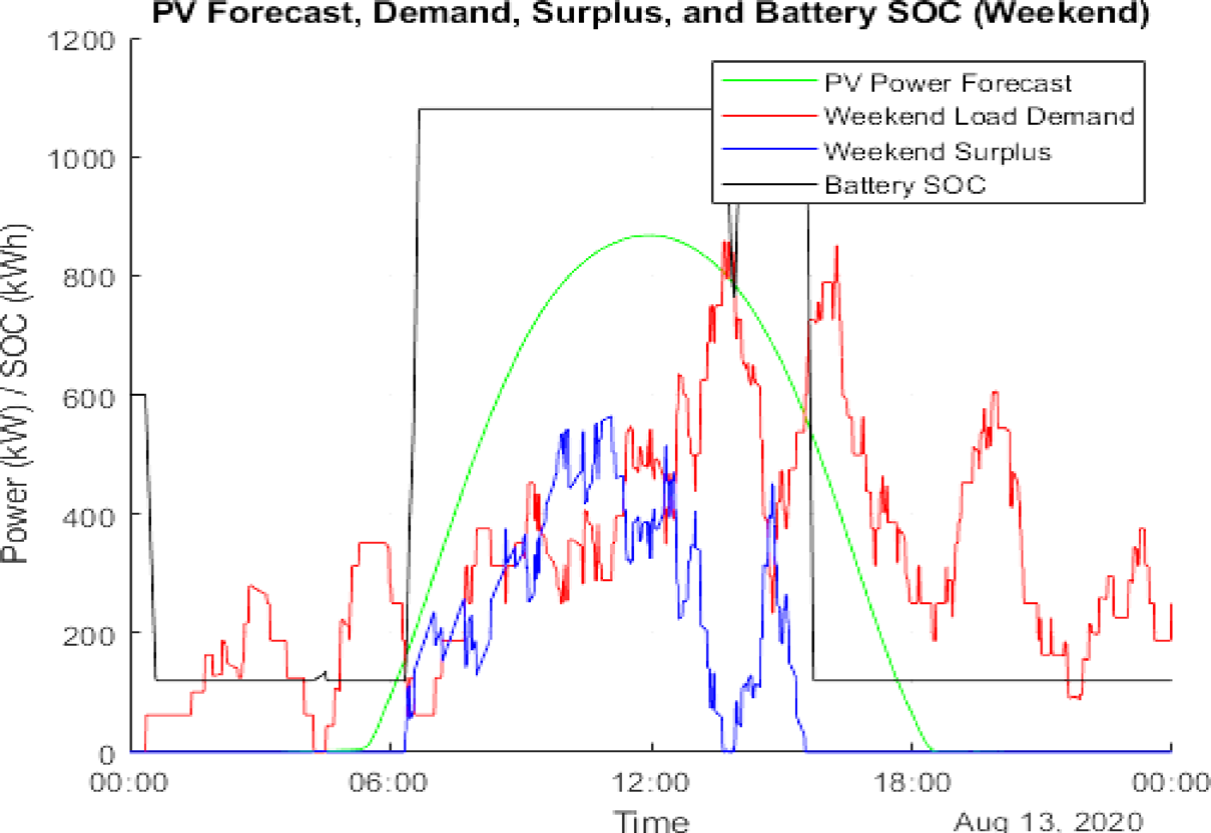
Weekend power surplus with PV and BESS.

Figure 13 visualizes the distribution of surplus and stored energy, while Fig. 14 tracks the daily battery State-of-Charge (SOC). These results demonstrate enhanced energy utilization and lower grid dependency, particularly during periods of high solar output.

### Energy reliability assessment

To evaluate the operational robustness and grid independence of the proposed UFCS system, two key reliability metrics were employed:

- Energy Sufficiency Ratio (ESR): The ESR represents the proportion of total energy demand that is fulfilled by local renewable sources — namely, solar PV and the battery energy storage system (BESS). It reflects how much of the load is met without relying on the utility grid.

Weekday: 57.4%.

Weekend: 78.95%.

These results indicate that during weekdays, over half of the UFCS’s energy needs are supplied by the integrated PV + BESS system. On weekends, the ratio increases substantially due to lower demand and higher availability of surplus solar generation.

- Autonomy Ratio (AR): The Autonomy Ratio measures the percentage of operating time during which the UFCS can run entirely without drawing any energy from the grid. It provides a measure of full grid independence.

Weekday: 59.03%.

Weekend: 51.74%.

While ESR is higher on weekends due to reduced load, the AR is slightly lower compared to weekdays. This is because short periods of high demand or low PV output — even if brief — can require grid support and reduce autonomy, even though the overall energy consumption is low.

#### Optimization and economic result

This section consolidates the optimization outcomes across all the three system configurations – Grid-only, PV-only, PV and BESS and interprets their techno-economic implications. The Genetic Algorithm (GA) was implemented in MATLAB to maximize Net Present Value (NPV) over a 20-year horizon, considering forecasted energy demand (weekday/weekend), GRU-based PV generation, capital and operational expenditures, and system constraints. Key input parameters included PV and BESS capacities, financial data (CAPEX, OPEX, replacement costs), and forecasted demand.

The results of the GA optimization are summarized in Table 3. In the baseline scenario, the UFCS is entirely dependent on grid-supplied electricity. The forecasted daily demand amounts to 9669.52 kWh on weekdays and 7776.41 kWh on weekends, leading to an annual energy consumption of approximately 3.33 GWh. The first-year revenue from EV charging operations is estimated at €2,332,743.58. However, the annual energy purchase cost from the grid totals €581,852.90, a significant operational expense that escalates over time.

Over the 20-year life cycle, the system yields a total Net Present Value (NPV) of €27.29 million. This scenario serves as a reference benchmark and illustrates the economic limitations of a grid-only UFCS model, which is highly exposed to electricity price volatility, grid congestion, and future carbon pricing. The lack of energy autonomy also poses risks related to grid failures and peak-hour tariffs. While simple to implement, this model lacks sustainability and long-term economic competitiveness.

**Table 3:** Results of optimization.

System Configuration	Optimal PV Size (kW)	BESS Capacity (kWh)	Maximized NPV (€)	NPV Improved (€)
Without PV and BESS	N/A	N/A	27.29	N/A
PV Only	999.98	N/A	33.48	+ 6.20
PV + BESS	1199.9	500.057	33.97	+ 6.68

### PV-Only configuration

In this configuration, the UFCS incorporates an optimized photovoltaic (PV) system to offset grid dependency. The simulation, guided by GRU-based solar forecasting, identifies an optimal PV sizing of approximately 1000 kW. Under this configuration, the system generates a higher NPV of €33.48 million, reflecting a profit gain of €6.19 million over the baseline grid-only scenario.

PV integration contributes significantly to reducing grid electricity purchases, especially during daytime hours. This results in considerable cost savings and a cleaner energy profile. However, due to the temporal mismatch between solar generation (peaking midday) and EV charging demand (spread throughout the day and evening), a substantial amount of generated PV energy is unutilized. On representative days (August 13 and August 9), surplus PV energy exceeds 90 MWh and 78 MWh respectively, highlighting inefficiencies. Despite these energy losses, the PV-only system offers a compelling economic improvement over the baseline. However, the findings also reveal the need for energy storage to manage surplus generation and further improve system utilization.

### PV and BESS configuration

The PV + BESS configuration integrates battery storage to address the limitations of the PV-only model. The GA-based optimization recommends a PV capacity of 1199.9 kW and a BESS capacity of 500.057 kWh. The battery stores excess solar energy generated during low-demand hours and discharges it during evening peaks or cloudy intervals, thereby smoothing the demand-supply gap.

The simulation results show that on weekdays, 358,308.80 kWh of PV energy is stored, while 354,529.78 kWh is stored on weekends. Remaining surplus energy is significantly reduced to 89,956.64 kWh (weekday) and 77,213.23 kWh (weekend), indicating a substantial improvement in renewable energy utilization. Grid electricity demand is also reduced to 165,598.63 kWh (weekday) and 96,103.16 kWh (weekend).

**Fig. 15:**
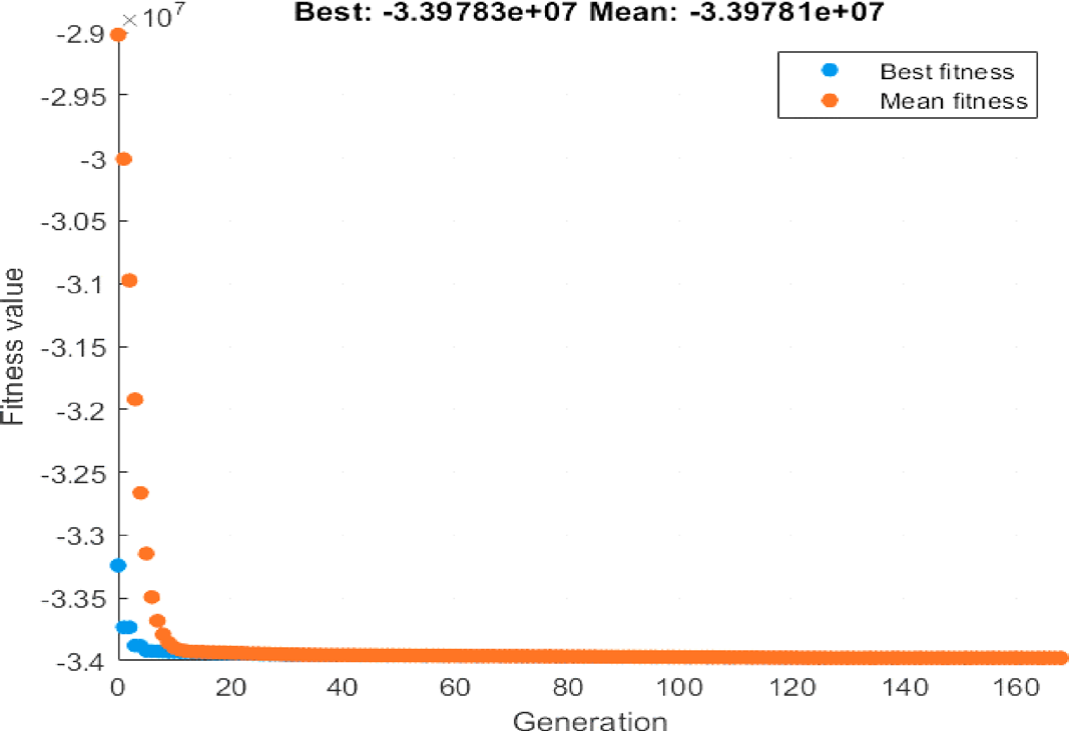
Convergence Curve of GA.

Financially, this scenario achieves a total NPV of €33.97 million, yielding an additional gain of €0.49 million over the PV-only configuration and €6.68 million over the baseline. The integration of BESS leads to enhanced energy reliability, lower curtailment, and greater autonomy. Figure 15 illustrates the GA convergence trend, confirming effective algorithm performance and rapid fitness convergence. Although BESS increases capital cost, it improves both technical and financial resilience, especially under dynamic solar availability.

### Sensitivity analysis

To evaluate the robustness of the proposed system under evolving market conditions, a sensitivity analysis was conducted. The cost parameters for sensitivity analysis are given in Table 4.

**Table 4:** Cost parameters for sensitivity Analysis.

Year	CAPEX PV (€/kW)	CAPEX BESS (€/kWh)	BESS replacement (€/kWh)
2020	1000	439	196
2025	900	380	159

It considers anticipated reductions in PV and battery costs based on industry trends. The new economic assumptions are as follows:

- PV CAPEX: €900/kW.
- BESS CAPEX: €380/kWh.
- BESS replacement cost: €159/kWh.

With these parameters, the GA optimization identifies a slightly adjusted optimal configuration of 1199.87 kW for PV and 505.47 kWh for BESS. This cost scenario results in an NPV of €34.05 million—an increase of €6.76 million compared to the baseline grid-only system and €80,000 above the current-cost PV + BESS configuration.

**Fig. 16:**
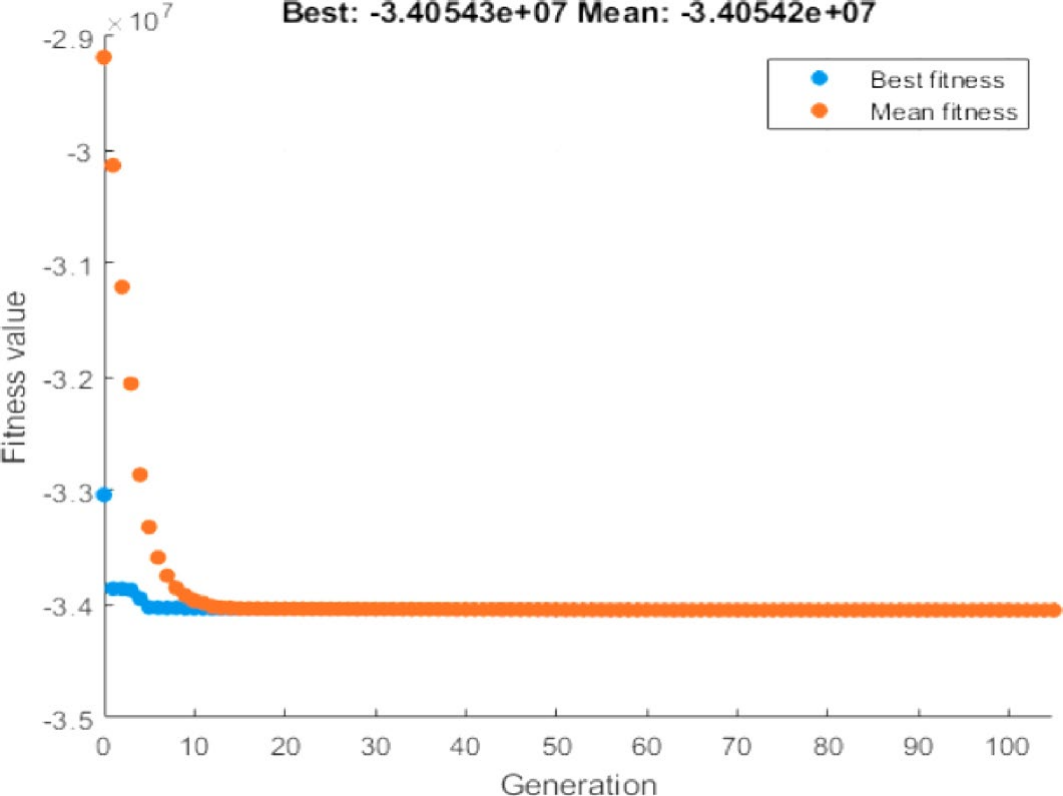
Convergence Curve of GA for sensitivity analysis.

Figure 16 displays the enhanced convergence behaviour under the revised cost structure, confirming the continued effectiveness of the GA in identifying profitable configurations. These results demonstrate that as component prices decline, the economic attractiveness of hybrid renewable UFCS systems will continue to grow, making them a sustainable long-term investment strategy.

### Discussion

The results of this study demonstrate the technical and economic benefits of integrating renewable energy systems into Ultra-Fast Charging Stations (UFCS) through a deep learning–driven optimization framework. Three configurations were evaluated: grid-only, PV-only, and PV + BESS, each optimized to maximize long-term Net Present Value (NPV). In the grid-only baseline, the UFCS achieves an NPV of €27.29 million over 20 years. This scenario serves as a reference, highlighting full reliance on grid energy and the associated high operational cost. While simple to deploy, it exposes the system to grid price volatility and peak load stress.

Scenario 1 (PV Integration) shows a notable improvement, with NPV rising to €33.48 million. This gain of €6.19 million confirms that PV integration significantly reduces grid dependency and improves cost-effectiveness. However, a large portion of the generated solar energy remains unutilized due to the mismatch between generation timing and demand, especially on weekdays. This underutilization presents a clear opportunity for storage optimization.

Scenario 2 (PV + BESS Integration) addresses this gap. By incorporating a 500.057 kWh battery, surplus solar energy is stored and used during peak demand or low-generation periods. This results in a higher NPV of €33.97 million — an additional gain of €490,000 over the PV-only case. The system benefits from smoother demand-supply balance, lower curtailment losses, and reduced energy imports from the grid.

The Energy Sufficiency Ratio (ESR) and Autonomy Ratio (AR) further validate the benefits of hybridization. ESR reaches 57.4% on weekdays and 78.95% on weekends, showing that most energy needs are met internally. AR values of 59.03% (weekday) and 51.74% (weekend) demonstrate that the UFCS can operate grid-independently for over half the time. These metrics confirm the technical viability of a renewable-powered UFCS, especially during periods of lower demand such as weekends.

Sensitivity analysis reveals that with projected reductions in PV and BESS CAPEX (to €900/kW and €380/kWh respectively), the optimized system achieves a peak NPV of €34.05 million. This result reflects a gain of €6.76 million over the baseline and indicates that the system becomes even more economically attractive as component costs continue to decline.

Overall, the PV + BESS configuration emerges as the most robust and financially viable option. The integration of deep learning–based solar forecasting (GRU), tailored weekday/weekend demand modeling, and evolutionary optimization (GA) enables a realistic, resilient, and profitable system design for UFCS — directly addressing both grid stress and sustainability objectives.

### Comparative analysis and novelty

The proposed framework demonstrates significant advancements over existing studies on renewable-powered Ultra-Fast Charging Stations (UFCS). While previous works have explored PV and BESS integration or generic optimization methods, they often rely on static demand profiles, traditional forecasting models, or simplified economic analysis. In contrast, this study delivers a holistic, data-driven approach that combines machine learning, system design, and financial modelling.

Key differentiators of this work include:

- Forecasting-Integrated Optimization: Unlike conventional models that assume average solar output, this study employs Gated Recurrent Unit (GRU) neural networks to generate high-resolution PV forecasts. These forecasts are directly integrated into the sizing optimization, ensuring that system capacities are aligned with temporal solar variability.
- Dual Demand Profile Modeling: This study is among the first to distinctly model weekday and weekend EV charging demand based on probabilistic vehicle arrival distributions. This differentiation reflects real-world usage patterns and enables more accurate energy planning for UFCS deployments.
- Economic Optimization Using NPV: The optimization framework maximizes Net Present Value (NPV) over a 20-year horizon by considering detailed CAPEX, OPEX, battery degradation, and energy trading dynamics. This contrasts with prior studies that often focus on short-term costs or energy efficiency alone.
- System Reliability Metrics: To assess operational independence and resilience, the study introduces Energy Sufficiency Ratio (ESR) and Autonomy Ratio (AR). These metrics quantify the system’s ability to operate without grid support—an aspect rarely addressed in comparable works.
- Forward-Looking Sensitivity Analysis: A parametric cost analysis evaluates how future reductions in PV and BESS costs affect profitability, offering valuable insights for long-term infrastructure planning and investment decisions.

Compared to earlier literature, this work provides a comprehensive and adaptive design strategy that reflects both technical and economic realities. By uniting deep learning-based forecasting, realistic demand modelling, and economic optimization, the proposed framework presents a replicable solution for cost-effective, high-performance UFCS design. Collectively, these innovations advance the state of the art in intelligent EV infrastructure design and planning.

## Conclusion

This study presents a comprehensive optimization framework for integrating photovoltaic (PV) and battery energy storage systems (BESS) into ultra-fast electric vehicle charging stations (UFCS). By employing deep learning-based solar forecasting—specifically using GRU and LSTM architectures—combined with a Genetic Algorithm (GA) for economic optimization, the proposed approach effectively maximizes the Net Present Value (NPV) while enhancing system reliability and reducing grid dependency. Simulation outcomes indicate that PV integration alone improves the NPV by €6.19 million over a 20-year evaluation period. When both PV and BESS are incorporated, the NPV increases to €33.97 million, achieving a €6.68 million improvement relative to a grid-only configuration. With future reductions in component costs considered, the optimized system configuration yields a maximum NPV of €34.05 million, representing a total enhancement of €6.76 million over the baseline.

The incorporation of GRU-based forecasting facilitates accurate, time-resolved sizing of PV and BESS capacities, ensuring demand-supply balance. Furthermore, reliability indicators such as Energy Sufficiency Ratio (ESR) and Autonomy Ratio (AR) affirm the hybrid system’s ability to support charging demand with reduced reliance on grid electricity—particularly during weekends with lower peak loads.

In summary, the results confirm that a hybrid PV-BESS integrated UFCS not only delivers significant economic returns but also advances environmental sustainability. This work underscores the importance of intelligent forecasting and optimization techniques in designing resilient and future-ready EV charging infrastructure.

### Future scope

Future research can explore the integration of emerging technologies such as high-efficiency photovoltaic modules and advanced energy storage systems, including solid-state and graphene-based batteries, to further enhance system performance and economic viability. As capital expenditures (CAPEX) for PV and BESS continue to decline due to technological advancements and policy incentives, large-scale deployment of renewable-powered UFCS will become increasingly feasible. Additionally, incorporating artificial intelligence (AI) and advanced machine learning algorithms for real-time forecasting and dynamic energy management can significantly improve operational efficiency, reduce costs, and support adaptive control strategies in varying load and weather conditions.

## Data Availability

Data Availability: The datasets used and/or analysed during the current study available from the corresponding author on reasonable request.
